# Gender, Sexuality, Mental Health, and Sexual Well-Being: Charting Burdens, Pathways, and an Affirmative Research Agenda

**DOI:** 10.3390/healthcare14131945

**Published:** 2026-07-01

**Authors:** Felipe Alckmin-Carvalho, Henrique Pereira

**Affiliations:** 1 Department of Psychology and Education, Faculty of Social and Human Sciences, University of Beira Interior, Pólo IV, 6200-209 Covilhã, Portugal; hpereira@ubi.pt; 2 Research Center in Sports Sciences, Health Sciences and Human Development (CIDESD), 5001-801 Vila Real, Portugal; 3 RISE-Health, Department of Psychology and Education, Faculty of Social and Human Sciences, University of Beira Interior, 6200-209 Covilhã, Portugal

## 1. Introduction

Gender, sexuality, mental health, and sexual well-being were, until recently, a marginal research topic. Over the past two decades, they have become a central concern in public mental health. The shift rests on a finding that population-based studies have reproduced many times: people whose sexual orientation or gender identity falls outside dominant cisgender and heterosexual norms experience higher burdens of depression, anxiety, suicidal behavior, and related mental-health difficulties than their cisgender heterosexual peers [1,2,3,4]. Research has since moved past simply documenting these disparities. It now asks why they arise, how they accumulate over a life, which social and political conditions deepen or ease them, and which interventions reduce harm rather than reproduce it.

Minority Stress Theory [5] is still the main framework for explaining these disparities. In Meyer’s account, distal stressors such as discrimination and violence, together with proximal stressors such as concealment, the expectation of rejection, and internalized stigma, convert social conditions into poor mental-health outcomes. Later work pushed the model past the interpersonal level. Stigma is also structural, embedded in laws, institutions, public policy, social and religious norms, and the uneven availability of affirming resources, and at this level, it is now recognized as a measurable determinant of mental, behavioral, and physical health among LGBTQIA+ populations [6,7,8]. The effect is to move the explanation away from identity and toward the environments in which people live.

This Special Issue came together during a tense period. Legal and policy disputes over LGBTQIA+ rights and gender-affirming care sharpened in several countries, and in Brazil, annual monitoring kept recording lethal anti-trans violence [9,10]. The harm is not only local: across countries, structural stigma predicts depression and suicidality among sexual minority men even after individual characteristics are taken into account, with internalized homonegativity and social isolation among the pathways involved [11]. The questions this collection takes up are clinical, social, and political at once.

LGBTQIA+ populations are not internally uniform, and intersectionality is the framework that keeps this in view. Crenshaw [12] introduced the concept; Bowleg [13] later brought it into public health. Exposure to stigma and access to affirming resources both vary with race, ethnicity, income, disability, migration status, religion, age, geographic context, and life stage. The studies gathered here cannot map every one of these intersections, but they point to several settings where a single-axis explanation falls short.

These frameworks emphasize risk, but risk is not the whole account. A complementary perspective, that of Minority Strengths, draws attention to identity pride, community connectedness, belonging, and collective meaning as resources that actively support resilience and well-being [14,15]. It does not displace Minority Stress Theory so much as work alongside it: discrimination is real, yet minority lives should not be read only through deficit and vulnerability. Several articles in this Special Issue show affirmation, community, and self-determination acting as protective resources, a thread we return to later.

This Special Issue gathers 12 empirical articles published in *Healthcare* between April 2025 and May 2026 [16,17,18,19,20,21,22,23,24,25,26,27], from Australia, Brazil, Greece, Poland, Portugal, Türkiye, the United Kingdom, and the United States. The quantitative articles are cross-sectional, drawing on group comparisons, regression, and mediation; the qualitative ones use secondary constructivist grounded theory [21], interpretive phenomenological analysis with autophotography [23], reflexive and codebook thematic analysis [17,19], and critical feminist poststructuralist inquiry [18]. Their participants are wide-ranging: female nurses [16], trans people navigating domestic and intimate partner violence services [17], patients with vaginismus [18], LGBT people with disabilities [19], transgender and gender-diverse adults [20], LGBTQ+ adults navigating healthcare [21], lesbian, gay, and bisexual students [22], trans men seeking gender-affirming medical care [23], LGBTQ+ adults exposed to anti-LGBTQ+ legislative debates [24], HIV pre-exposure prophylaxis (PrEP) users [25], women across the reproductive life cycle [26], and cisgender gay men [27]. Read together, they show how mental and sexual health take shape across clinical, occupational, relational, legal, and institutional settings.

Although several contributions focus specifically on LGBTQIA+ populations, the issue also includes studies on women’s sexual and occupational well-being, reflecting the broader aim of examining how gendered and sexual health experiences are shaped by clinical, occupational, institutional, and relational contexts. Hence, this Special Issue argues that mental health and sexual well-being among gender and sexually diverse populations cannot be understood through individual-level risk alone; they must be examined as outcomes of structural stigma, intersecting inequalities, clinical normativity, and the presence or absence of affirming resources.

## 2. An Overview of the Studies in This Special Issue

Table 1 lists the 12 articles by date of publication, with their objectives, methods, main findings, and implications. It maps the collection and sets up the thematic synthesis that follows.

Most of the studies draw on convenience, purposive, snowball, or service-based samples. That is often unavoidable when the populations are marginalized or clinically specific, but it limits what can be inferred at the population level. The quantitative work is cross-sectional, using group comparisons, regression, and mediation; the qualitative work ranges from grounded theory and phenomenology to thematic analysis and critical feminist poststructuralism. The mix matters because prevalence, mechanism, lived experience, and institutional practice each call for a different kind of evidence.

The thematic synthesis below was developed by reading the 12 contributions comparatively and identifying recurring conceptual, methodological, and practice-oriented issues across studies.

## 3. Cross-Cutting Themes

### 3.1. Minority Stress, Structural Stigma, and Political Climate

Minority stress is the clearest theoretical thread across the collection. Watts et al. [24] extend the concept of structural stress by showing that legislative debate, not only enacted law, may become a psychologically harmful exposure. In their Kentucky sample, substantial proportions of LGBTQ+ adults attributed increased suicidal thoughts or a suicide attempt to anti-LGBTQ+ legislative debates. This finding aligns with cross-national evidence linking structural stigma to depression and suicidality [11] and with broader reviews of structural stigma and LGBTQ+ health [7,8].

Other studies locate minority stress in more proximal settings. Ribeiro-Gonçalves et al. [22] found that Portuguese LGB university students reported lower optimism, higher self-reported academic performance, and lower DASS-21 symptom scores than heterosexual students, a pattern that resists a uniform deficit interpretation and may reflect compensatory or resilience-related processes. Alckmin-Carvalho et al. [27] show that psychological distress among Brazilian gay men also varies with socioeconomic position and racialized exposure to stigma. Taken together, the studies trace minority stress across public debate, institutions, families, universities, and everyday interaction.

### 3.2. Intersectionality and Compounded Stigma

Several articles demonstrate the limits of single-axis accounts of sexual and gender minority mental health. Alckmin-Carvalho et al. [27] found higher trait and state anxiety among Black and brown Brazilian gay men than among white gay men, without corresponding group differences in depression or homonegativity. This outcome-specific pattern underscores that intersecting stigmas need not affect every dimension of distress uniformly.

Soares et al. [19] and Andrade and Costa [17] make complementary institutional arguments. In disability services, sexual orientation and gender identity may become invisible when disability is treated as the only clinically relevant axis. In domestic and intimate partner violence services, trans people may fall between generalist systems with limited trans competency and LGBTQIA+ services that are not equipped to address violence. Pithavadian et al. [18] extend this critique to vaginismus assessment, showing how heteronormativity, cisnormativity, ethnocentricity, chrononormativity, and able-bodied normativity shape what clinicians ask, notice, and define as treatment success. Together, these studies reinforce the need to translate intersectionality into both population health methods and routine clinical practice [13,28].

### 3.3. Healthcare Access, Provider Competency, and Affirming Care

Access to healthcare cannot be reduced to the presence or absence of a service. Gillani et al. [21] show that LGBTQ+ people undertake substantial work to preserve or reconstruct a sense of wholeness within systems that may affirm or fragment identity. Cosford and Williamson [23] similarly identify progression through gender-affirming care as a major protective resource for British trans men, whereas delays, rejection, and hostile social climates undermine well-being. Across these studies, professional goodwill is valuable but insufficient when organizations lack appropriate competencies, pathways, and accountability [17,19,21].

Two studies not centered on LGBTQIA+ populations broaden this discussion through a gender lens. Valetta et al. [16] link work-related quality of life, mental health, and sexual functioning among female nurses, whose own well-being may be overshadowed by the demands of care delivery. Kırat [26] shows that the relationship between sexual function and psychological distress varies across women’s reproductive life stages. Read alongside the LGBTQIA+-focused contributions, these studies demonstrate that gendered determinants of mental and sexual health operate across populations and become particularly consequential where clinical invisibility, stigma, or exclusion is present.

### 3.4. Protective Factors: Belonging, Affirmation, and Meaning

Protective factors in this collection are more than the absence of risk. Community belongingness was associated with lower odds of legislative debate-attributed suicidal thoughts and suicide attempts in Watts et al. [24]. Cosford and Williamson [23] identified acceptance, belonging, connection with nature, and access to gender-affirming care as resources for trans men. Gillani et al. [21] placed intra-community support, self-determined care, and meaning-finding at the center of a model of wholeness, while Ribeiro-Gonçalves et al. [22] identified optimism as a predictor of well-being among LGB students.

These findings accord with the Minority Strengths perspective [14,15]. Pride, belonging, affirmation, and meaning are not merely individual coping styles; they are produced through relationships, communities, and institutions. Resilience language should therefore not transfer responsibility for structural harm back to marginalized people. Protective resources matter because they may help buffer harms that remain social, institutional, and political [5,11,29].

## 4. Implications for Research, Practice, and Policy

In this editorial, sexual well-being is understood broadly, encompassing sexual function, safety, autonomy, pleasure, relational context, access to affirming care, and freedom from coercion, stigma, and normative clinical assumptions. Across several contributions, clinical harm is not produced only by overt discrimination, but also by taken-for-granted assumptions about bodies, relationships, sexual scripts, and treatment goals.

Therefore, the collection points to four research priorities. First, sexual orientation and gender identity should be collected routinely, confidentially, and with appropriate response options in clinical, educational, and population health systems; without such data, inequities remain difficult to document and address [4,30]. Second, longitudinal, multilevel, and cross-national designs are needed to distinguish structural, institutional, interpersonal, and individual pathways [7,11]. Third, quantitative intersectional methods—including Multilevel Analysis of Individual Heterogeneity and Discriminatory Accuracy (MAIHDA) [31], latent class approaches, and adequately powered subgroup analyses—should be used more widely when the goal is to translate intersectional theory into population-level evidence. Fourth, research must better include understudied identities, life stages, and regions, including intersex, asexual, aromantic, nonbinary, older, disabled, and migrant LGBTQIA+ populations and settings in Africa, Asia, the Middle East, and Latin America beyond Brazil.

Measurement warrants equal attention. Many instruments used in this field were developed primarily with cisgender heterosexual samples. This does not make them unusable, but it means that measurement equivalence and cultural validity cannot be assumed. Pithavadian et al. [18] illustrate the problem in vaginismus assessment, where conventional endpoints may treat penile–vaginal intercourse as the implicit standard of success. Comparable scrutiny is needed when measures of depression, trauma, sexual function, and quality of life are applied across gender-diverse and culturally diverse populations.

In clinical practice, trauma-informed and gender-affirming approaches are particularly important for transgender and gender-diverse people whose mental-health trajectories may be shaped by childhood adversity, family rejection, delays in care, and structural stigma [20,23]. Respectful language is necessary but does not by itself create affirming care. Organizations also require trained staff, clear care and referral pathways, supervision, accountability, and outcome monitoring tailored to the populations they serve [17,19,21]. The Standards of Care for the Health of Transgender and Gender Diverse People, Version 8 [32] remains an important reference for transgender-specific clinical work.

Competency in gender and sexual diversity is also needed across adjacent fields. Gynecology, nursing, primary care, occupational health, disability services, and domestic violence services appear in this Special Issue as settings in which mental health, sexual health, and institutional practice intersect [16,17,18,19,26]. Diversity should therefore be embedded in routine assessment, communication, care planning, and evaluation rather than added as an afterthought.

The policy implications are direct. Watts et al. [24] associate legislative debate with suicidality, while Pachankis et al. [11] link country-level structural stigma with depression and suicidality among sexual minority men. Legal and policy environments are not neutral background conditions; they can act as determinants of population mental health. This perspective has consequences for the conduct of public debate, the protection of evidence-based gender-affirming care, the resourcing of community organizations, and the response of healthcare systems when public policy itself becomes a source of harm.

Institutional policies should also reduce the burden of self-advocacy placed on LGBTQIA+ people. Inclusive documentation, confidential data collection, transparent referral systems, staff accountability, and sustained partnerships with community organizations are practical measures. At the population level, anti-discrimination protections and investment in accessible, affirming services should be understood not only as statements of principle but also as components of mental-health prevention.

The Special Issue also has limitations. Most studies rely on cross-sectional or qualitative designs, and several use non-probability samples. Geographic coverage remains uneven, with limited representation from Africa, Asia, the Middle East, and Latin America beyond Brazil. Some identities, including intersex, asexual, aromantic, nonbinary, older, disabled, migrant, and racialized LGBTQIA+ populations, remain underrepresented. These limitations should guide, rather than diminish, the next phase of research.

## 5. Conclusions

Sexual and gender minorities continue to experience a disproportionate burden of mental-health difficulties, but that burden is not intrinsic to identity. It is patterned by minority stress, structural stigma, intersecting inequalities, developmental adversity, healthcare exclusion, and the availability, or absence, of affirming resources.

The studies in this Special Issue move the field beyond prevalence estimates toward a more integrated account of social, institutional, developmental, and protective processes. They show that hostile political climates, clinical normativity, and fragmented services can produce harm, while belonging, affirming care, inclusive assessment, self-determination, and meaning can support well-being. The task ahead is to convert this knowledge into measurable changes in healthcare, education, workplaces, social and legal services, and community settings. The ultimate goal is not only to reduce risk but to create conditions in which sexual and gender minorities can live dignified, valued, and flourishing lives.

We hope this collection will support researchers, clinicians, service leaders, and policymakers in designing systems that recognize gender and sexual diversity as part of the population for whom care is intended. An affirmative research agenda should therefore combine structural measurement, intersectional methodology, inclusive clinical assessment, community-based intervention, and policy accountability. Such an agenda asks not only how stigma harms, but also how institutions can actively produce safety, dignity, belonging, pleasure, and flourishing.

## Figures and Tables

**Table 1:** Synthesis of the 12 studies published in the *Healthcare* Special Issue “Gender, Sexuality and Mental Health”.

#/Title	Authors/Country/Year	Objective	Method	Main Findings and Implications
1. Work-Related Quality of Life, Mental Health, and Female Sexual Well-Being Among Nurses [16]	Valetta et al./Greece/2026	Examine work-related quality of life (WRQoL), mental health, and female sexual well-being among nurses in tertiary care.	Quantitative cross-sectional study of 100 female nurses at a tertiary general hospital in Greece. Measures: WRQoL scale, DASS-21, FSFI, and sociodemographic and occupational variables; Pearson correlations and multiple linear regression adjusted for age, marital status, number of children, and work experience.	Depression scores were negatively associated with sexual function (β = −0.388, *p* = 0.029), whereas stress scores were positively associated (β = 0.371, *p* = 0.038); the model explained 18.6% of the variance. The findings link nurses’ occupational, psychological, and sexual well-being and support greater attention to staff well-being in gender-sensitive healthcare.
2. Trans* People Experiencing Domestic and Intimate Partner Violence: Insights from Professionals Within Portugal’s National Support Network [17]	Andrade and Costa/Portugal/2026	Explore how professionals in Portugal’s domestic violence support network understand and respond to trans people experiencing domestic and intimate partner violence.	Qualitative study using codebook thematic analysis of semi-structured interviews with eight professionals from Portugal’s National Support Network for Victims of Domestic Violence.	Participants described cumulative vulnerabilities, particularly among younger trans people, related to family and intimate partner violence, service gaps, limited specialized training, scarce culturally competent provision, and labor-market exclusion. The study supports trans-affirming training, referral pathways, and institutional protocols rather than reliance on professional goodwill alone.
3. Health Professionals’ Approaches to Support Patient Diversity in the Assessment of Vaginismus: A Critical Feminist Qualitative Study for Inclusive Care [18]	Pithavadian et al./Australia/2026	Explore health professionals’ experiences and perceptions of patient diversity in the assessment and support of people with vaginismus.	Qualitative study based on semi-structured interviews with 23 health professionals across six disciplines, analyzed through a critical feminist poststructuralist lens.	Sexuality, gender, age, disability, ethnicity, and religion were addressed unevenly and often filtered through heteronormative and able-bodied clinical scripts; reported treatment goals also differed by sexual orientation. Inclusive assessment should move beyond penile–vaginal intercourse as the default outcome and attend to diverse bodies, relationships, goals, and meanings of sexual well-being.
4. How Do Portuguese Care Providers Address Disability and LGBT Identity in Their Work? [19]	Soares et al./Portugal/2026	Understand Portuguese professionals’ attitudes and practices concerning sexuality and LGBT belonging among people with disabilities.	Qualitative study using reflexive thematic analysis of semi-structured interviews with 11 professionals, including psychologists, occupational therapists, and personal care assistants.	Limited knowledge of LGBT identities, cis-heteronormative assumptions, family management, dependence on others, and absent institutional guidance constrained affirmative practice. Services need institutional training, inclusive sexuality education, and explicit guidance so that disability does not eclipse sexual orientation and gender identity.
5. How Childhood Maltreatment Contributes to Explaining Depressive Symptoms in Transgender and Gender-Diverse Individuals [20]	Parker and Rogowska/Poland/2026	Test whether childhood maltreatment helps explain the association between gender identity and depressive symptoms among transgender and gender-diverse adults.	Quantitative cross-sectional online study of 249 adults (144 cisgender and 105 transgender or gender-diverse). Measures: PHQ-9 and CTQ-SF; group comparisons and parallel mediation models.	Transgender and gender-diverse participants reported higher depressive symptoms and higher levels of all maltreatment subtypes than cisgender participants. Childhood trauma partially mediated the association, with emotional abuse as the primary mediator and a smaller indirect effect for sexual abuse. The findings support trauma-informed, gender-affirming care without locating pathology in gender identity itself.
6. Constructing Wholeness in LGBTQ+ Healthcare Access: A Grounded Theory Model [21]	Gillani et al./United States/2026	Develop a grounded theory model of how LGBTQ+ people construct wholeness while navigating healthcare systems.	Qualitative secondary constructivist grounded theory analysis of data from a community-based system dynamics project involving 28 LGBTQ+ participants.	Healthcare systems alternately supported or fragmented identity, while participants responded through interconnecting selves, intra-community support, self-determined care, and meaning-finding. Affirming systems should support identity integration, community connection, self-determination, and meaning-making while reducing the burden of individual self-advocacy.
7. Well-Being and Sexual Diversity in Higher Education: The Role of Mental Health, Optimism, Academic Performance, and Motivation in Portuguese Students [22]	Ribeiro-Gonçalves et al./Portugal/2026	Assess well-being, mental-health symptoms, optimism, motivation, and academic performance by sexual orientation among Portuguese higher-education students.	Quantitative cross-sectional survey of 285 students (129 heterosexual and 156 LGB), aged 18–69. Measures included well-being, DASS-21 symptoms, optimism, academic motivation, and self-reported academic performance; group comparisons and regression analyses.	LGB students reported lower optimism, higher self-reported academic performance, and lower DASS-21 symptom scores (indicating better mental health) than heterosexual students. Optimism was the strongest predictor of well-being among LGB students, whereas the DASS-21 mental-health indicator was strongest among heterosexual students. The cross-sectional, self-report design warrants cautious interpretation.
8. An Autophotographic–Phenomenological Investigation of British Transmen’s Psychological Well-Being [23]	Cosford and Williamson/United Kingdom/2026	Explore how psychological well-being is threatened and supported among British trans men seeking gender-affirming medical care.	Qualitative interpretive phenomenological analysis combining online semi-structured interviews and autophotography with 11 trans men aged 18–68.	Participants described longstanding mental-health difficulties, while rejection, healthcare barriers, and hostile social climates undermined well-being. Acceptance, belonging, connection with nature, and progress in gender-affirming care were protective, supporting affirmative psychological care, accessible medical pathways, and community allyship.
9. Legislative Debate-Attributed Suicidality Among LGBTQ+ Adults: The Buffering Effect of Community Belongingness [24]	Watts et al./United States/2026	Examine whether LGBTQ+ community belongingness is associated with suicidality attributed to anti-LGBTQ+ legislative debates.	Quantitative secondary cross-sectional analysis of the 2025 Queer Kentucky Survey (N = 817; non-probability snowball sample). Binary logistic regression was adjusted for age, race, gender identity, education, and income.	In this sample, 59.7% attributed increased suicidal thoughts and 44.1% attributed a suicide attempt to anti-LGBTQ+ legislative debates. Community belongingness was associated with lower odds of ideation (OR = 0.61) and attempts (OR = 0.41), while transgender and BIPOC participants had higher odds. The findings support structural and intersectional responses while avoiding causal interpretation of cross-sectional data.
10. Sexual Orientation and Gender Identity Associated with Sexual Practices, Psychoactive Substance Use and Sexually Transmitted Infections Among HIV PrEP Users [25]	Silva et al./Brazil/2025	Analyze associations between sexual orientation, gender identity, sexual practices, recent sexually transmitted infections, and psychoactive substance use among PrEP users in São Paulo.	Quantitative cross-sectional analysis of clinical records from 736 HIV PrEP users at a São Paulo health service (2018–2021), using sociodemographic, sexual-behavior, STI, and substance-use variables and binomial logistic regression.	Condomless sex was recorded for 98.5% of users, 18.4% had a recent STI, and 55.4% reported psychoactive substance use. Transgender and cisgender women had higher odds of reporting sex work and crack use, while homosexual and bisexual participants had higher odds of erectile-stimulant use. Prevention should be tailored to clinically meaningful subgroup profiles.
11. Sexual Dysfunction in the Life Cycle of Women: Implications for Psychological Health [26]	Kırat/Türkiye/2025	Examine associations between sexual dysfunction, depression, anxiety, and stress among premenopausal, pregnant, and postmenopausal women.	Prospective cross-sectional study of 300 women aged 18–70 presenting with sexual-dysfunction symptoms to a tertiary gynecology outpatient clinic. Measures: FSFI and DASS-21; group comparisons and logistic regression.	Sexual dysfunction was most frequent among postmenopausal women (96%); lubrication and orgasm scores declined after menopause, severe stress was more prevalent among premenopausal women, and FSFI scores correlated weakly and negatively with DASS-21 total, anxiety, and depression. Parity was the only independent multivariable predictor, supporting individualized, life-stage-aware assessment.
12. Perceived Homonegativity and Psychological Distress in Gay Men in Brazil: Does Skin Color Matter? [27]	Alckmin-Carvalho et al./Brazil and Portugal/2025	Assess associations among skin color, perceived homonegativity, depressive and anxiety symptoms, and socioeconomic variables in Brazilian cisgender gay men.	Quantitative cross-sectional survey of 229 Brazilian gay men (151 white and 78 Black or brown). Measures: Internalized Homophobia Scale, BDI-II, and STAI; group comparisons and hierarchical linear regression.	Homonegativity, depressive symptoms, and trait and state anxiety were elevated, particularly among younger and lower-income participants. Black and brown participants reported higher anxiety than white participants, and skin color predicted anxiety but not depression or homonegativity. Interventions should address both sexual prejudice and racial discrimination.

Note: LGB = lesbian, gay, and bisexual; LGBT/LGBTQ+ are used according to the terminology of each original article; WRQoL = work-related quality of life; PHQ-9 = Patient Health Questionnaire–9; CTQ-SF = Childhood Trauma Questionnaire–Short Form; FSFI = Female Sexual Function Index; DASS-21 = Depression Anxiety Stress Scales–21; BDI-II = Beck Depression Inventory–II; STAI = State–Trait Anxiety Inventory; STI = sexually transmitted infection; PrEP = pre-exposure prophylaxis; BIPOC = Black, Indigenous, and People of Color; OR = odds ratio.

## Data Availability

No new data were created or analyzed in this editorial.
